# Supplementary material for: Helicobacter pylori and Campylobacter jejuni bacterial holocytochrome c synthase structure-function analysis reveals conservation of heme binding
Source: Commun Biol. 2024 Aug 13;7:984. doi: 10.1038/s42003-024-06688-3 (PMC11322641; doi:10.1038/s42003-024-06688-3)
Supplement: Supplementary file 2 — Supplementary Information [file 42003_2024_6688_MOESM2_ESM.pdf]

Supplementary Information

Title: *Helicobacter pylori* and *Campylobacter jejuni* bacterial holocytochrome *c* synthase structure-function analysis reveals conservation of heme binding

Authors: Tania Yeasmin[1], Susan C. Carroll[2], David J. Hawtof[2,3], Molly C. Sutherland[2,*]

[1]Department of Chemistry and Biochemistry, University of Delaware, Newark, DE 19716 USA
[2]Department of Biological Sciences, University of Delaware, Newark, DE 19716 USA
[3]Current Address: Department of Biology, University of Virginia, Charlottesville, VA 22904, USA
[*]Address correspondence to Molly C. Sutherland, msuther@udel.edu

Table of Contents

SUPPLEMENTARY FIGURE 1 3
SUPPLEMENTARY FIGURE 2 4
SUPPLEMENTARY FIGURE 3 5
SUPPLEMENTARY FIGURE 4 7
SUPPLEMENTARY FIGURE 5 8
SUPPLEMENTARY FIGURE 6 9
SUPPLEMENTARY FIGURE 7 10
SUPPLEMENTARY FIGURE 8 12
SUPPLEMENTARY FIGURE 9 14
SUPPLEMENTARY FIGURE 10 15
SUPPLEMENTARY FIGURE 11 17
SUPPLEMENTARY FIGURE 12 18
SUPPLEMENTARY METHODS 19
SUPPLEMENTARY TABLE 1. 20
SUPPLEMENTARY REFERENCES 24

Supplementary Figure 1

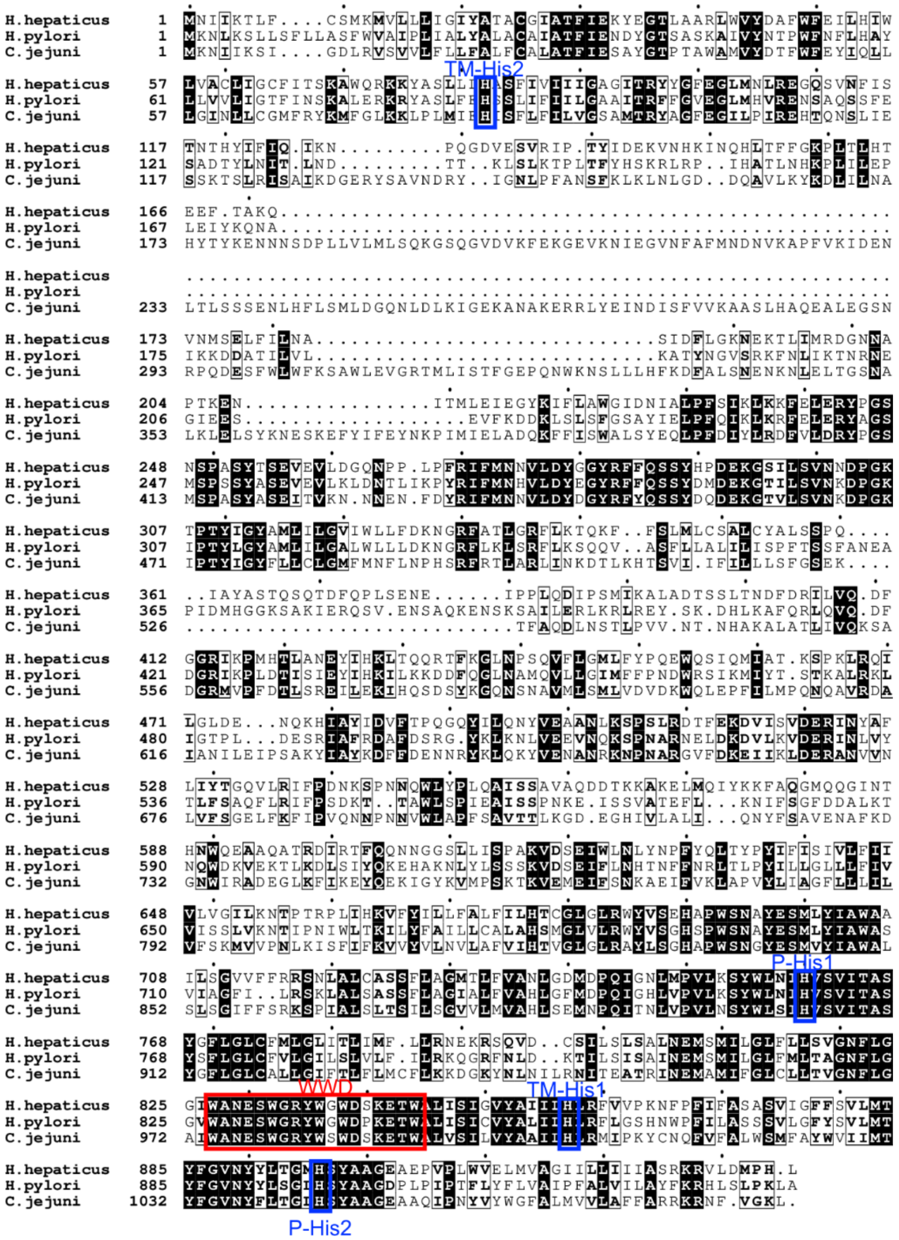

Supplementary Figure 1. Sequence alignment of *H. hepaticus*, *H. pylori* and *C. jejuni* CcsBA. Protein sequences (*H. hepaticus:* WP_041309336.1, *H. pylori:* WP_000793250.1 *C. jejuni:* CAL35131.1) were aligned using T-Coffee[1,2] and visualized with ESPRipt 3.0[3]. Residues highlighted in black are identical and residues outlined in black are similar. The conserved histidines are labeled and outlined in blue. The WWD domain is labeled and outlined in red.

Supplementary Figure 2

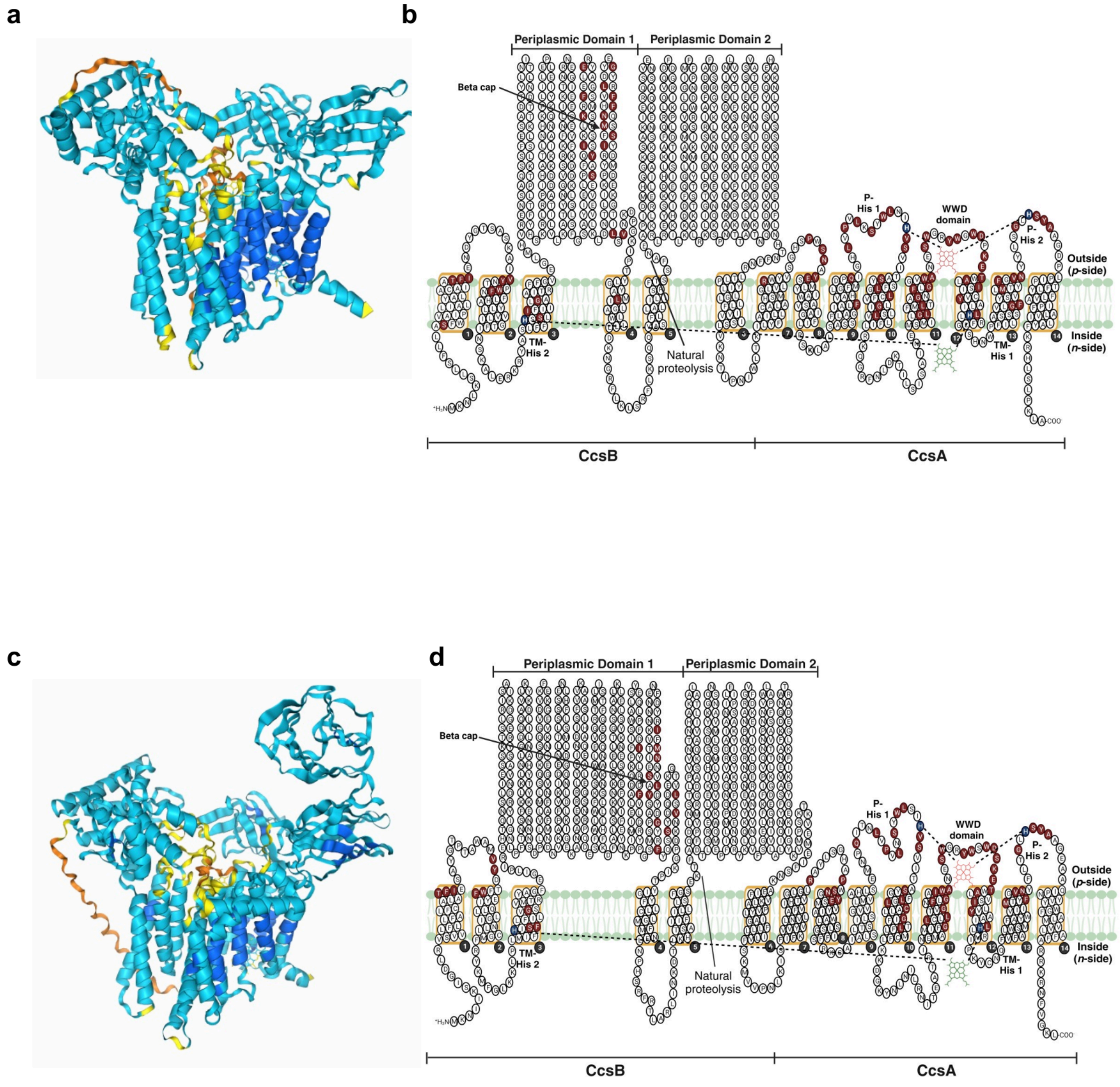

Supplementary Figure 2. Predicted AlphaFold structures and topologies of *H. pylori* and *C. jejuni* CcsBAs. a, c) AlphaFold 3[4] structural predictions were generated for (a) *H. pylori* CcsBA using protein sequence (WP_000793250.1) and c) *C. jejuni* CcsBA using protein sequence (CAL35131.1). The confidence measures are shown: dark blue, pLDDT>90; light blue, pLDDT 70-90; yellow, pLDTT 50-70; yellow pLDTT<50. b, d) Based on the *H. hepaticus* CcsBA Cryo-EM structure[5] and TMHMM topology predictions[6] the topologies of b) *H. pylori* CcsBA and d) *C. jejuni* CcsBA were created with BioRender.com (agreement numbers *UX26CDL17R, KB26CDLE1A*). The conserved histidines (blue), conserved residues (red) and WWD domain are labeled. The predicted natural proteolysis site and beta cap region are indicated.

Supplementary Figure 3

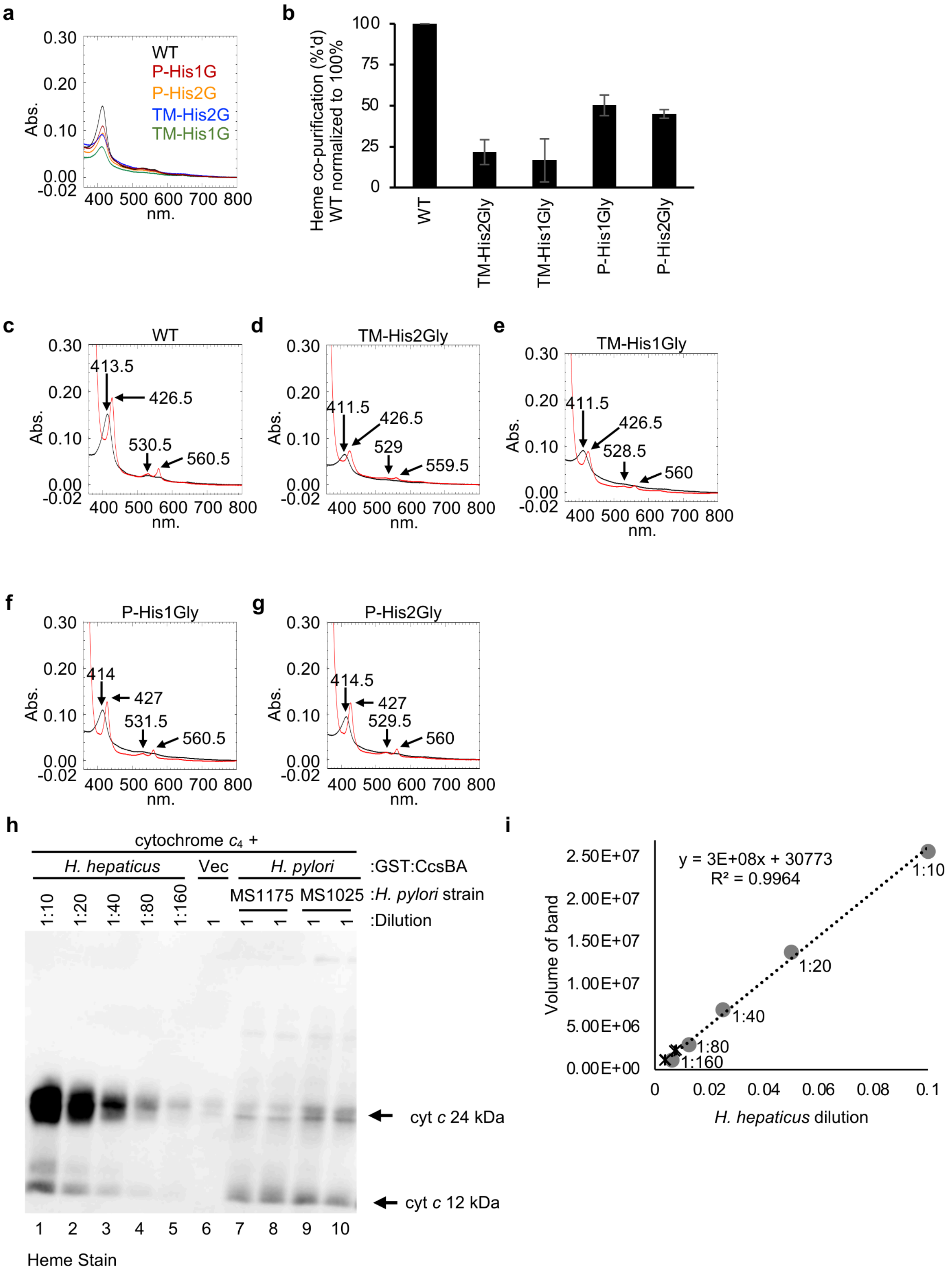

Supplementary Figure 3. *H. pylori* CcsBA His → Gly variant analysis. a-g) 75 µg of affinity purified protein was used for analysis. a,b) Quantification of heme co-purification was monitored via heme Soret (~412 nm). Soret analysis is representative of two, independent purifications. c-g) UV-vis spectroscopy of the indicated purified protein was determined. As purified (black) and reduced (red) spectra with key peaks indicated. UV-vis spectra are representative of three, independent purifications. h) Determination of *H. pylori* GST:CcsBA synthase function. Either *H. hepaticus* or *H. pylori* CcsBA was co-expressed with cytochrome c_4 *in E. coli* Δ*ccm.* Synthase activity (i.e. heme attachment to cytochrome c_4) was determined

by separation of total cell lysate via SDS-PAGE and heme stain. To determine the efficiency of *H. pylori* synthase function, dilutions of the functional assay performed with *H. hepaticus* CcsBA (1:10 – 1:160; lanes 1-5) were included. To ensure the low synthase activity of *H. pylori* was not due to a mutation in the recombinant *E. coli* strain, two independently constructed strains were used (MS1175, MS1025 – *E. coli* Δ*ccm* with cytochrome c_4 (pRGK334) and wild type *H. pylori* GST:CcsBA (pMCS1075)) and assay was run in duplicate (lanes 7/8, 9/10). i) Quantification of heme stained bands was performed and *H. hepaticus* dilution series (gray circles) was used to generate a standard curve (dashed line). The equation of the line and the volume of the cytochrome c_4 heme stained bands was used to determine the relative *H. pylori* CcsBA (black X) synthase function compared to *H. hepaticus* CcsBA. *H. pylori* CcsBA has ~1/175 function of *H. hepaticus* CcsBA across the 4 samples.

Supplementary Figure 4

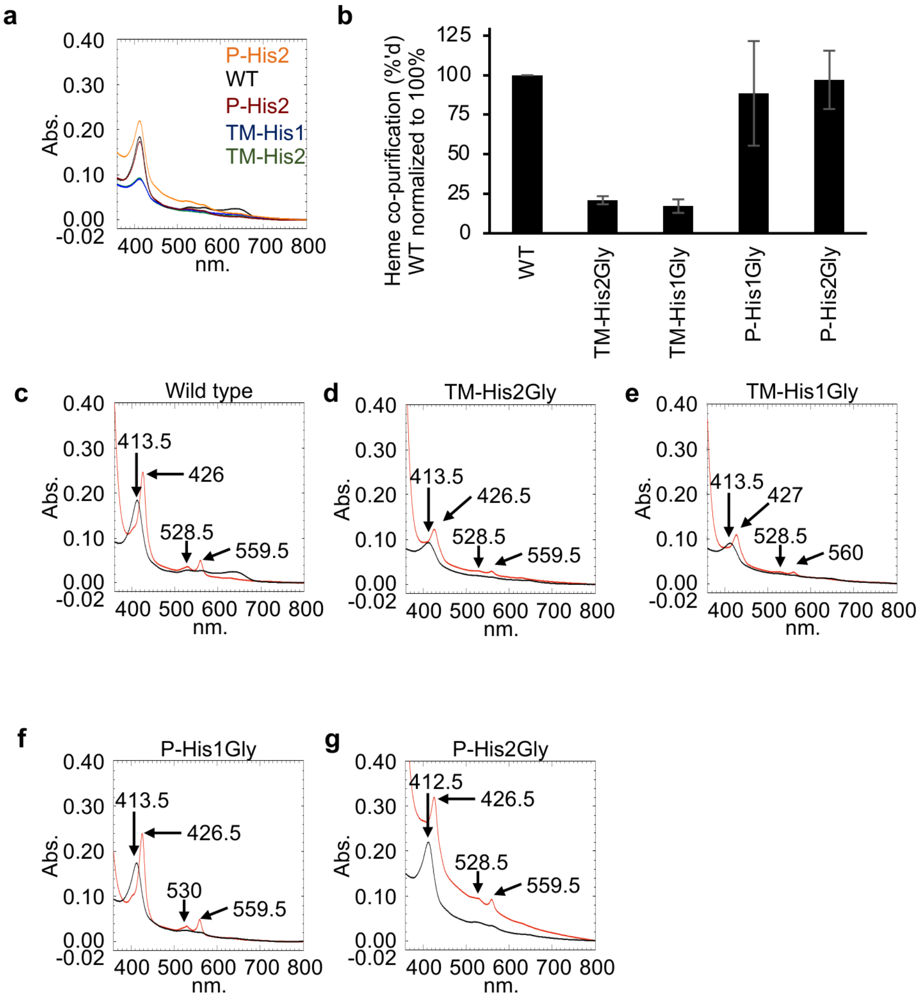

Supplementary Figure 4. UV-vis spectral analysis of *C. jejuni* CcsBA His → Gly variants. 75 µg of affinity purified protein was used for analysis. a, b) Quantification of heme co-purification was monitored via heme Soret (~412 nm). c-g) UV-vis spectroscopy of indicated purified protein was determined. As purified (black) and reduced (red) spectra with key peaks indicated are shown. Data are representative of three, independent purifications.

Supplementary Figure 5

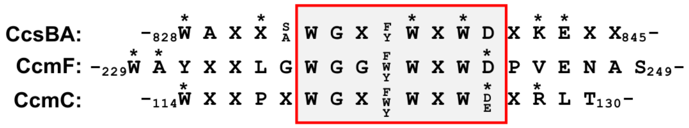

Supplementary Figure 5. Alignment of WWD domains from bacterial cytochrome *c* biogenesis proteins. The consensus sequence of WWD domain is shown with the highly conserved region boxed in red. Numbers indicate amino acid numbers from specific proteins that have previously been analyzed for formation of a cysteine/heme crosslink in *H. hepaticus* CcsBA[7], *E. coli* CcmF[8], *E. coli* CcmC[9].

Supplementary Figure 6

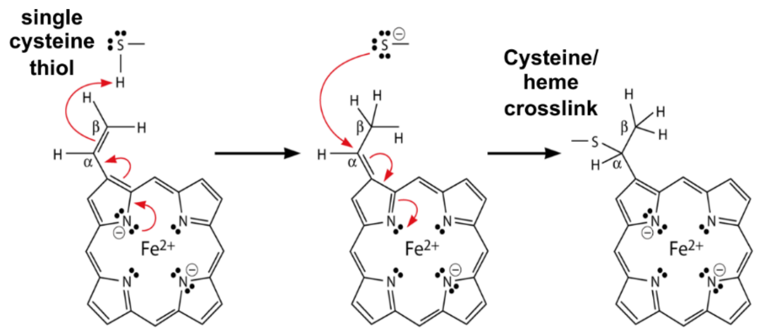

Supplementary Figure 6. Mechanism of cysteine/heme crosslink formation. A single cysteine thiol is shown for simplicity. Red arrows represent a 2-electron transfer. A covalent thioether bond is formed between the cysteine thiol and the alpha carbon of the heme vinyl group (Figure modified from[9] with permission from Elsevier, originally published in Journal of Molecular Biology Vol 430, Issue 8, Sutherland MC *et al*. Structurally Mapping Endogenous Heme in the CcmCDE Membrane Complex for Cytochrome c Biogenesis, pg 1065-1080, copyright Elsevier (2018)).

Supplementary Figure 7

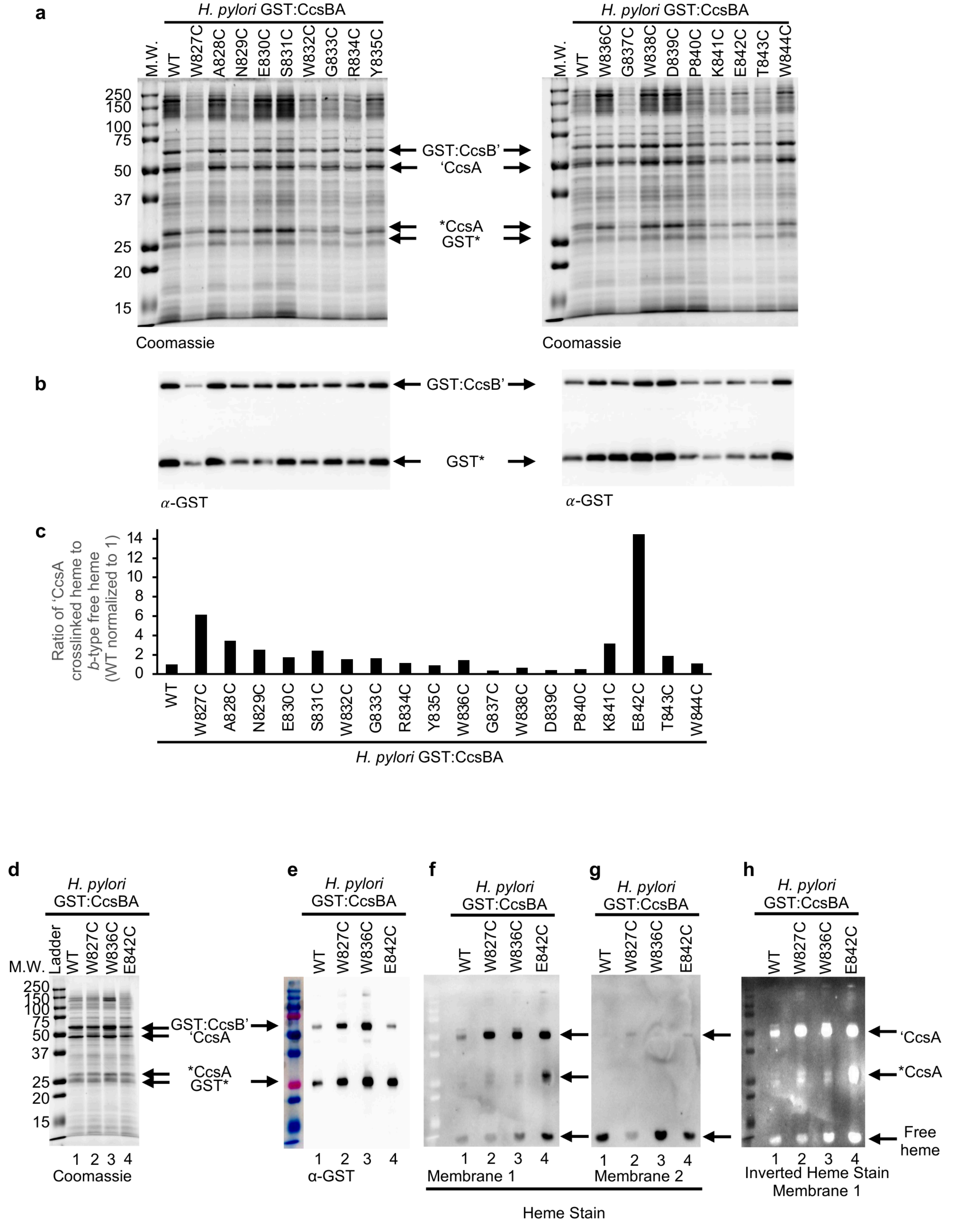

Supplementary Figure 7. Cysteine/heme crosslinking in the *H. pylori* CcsBA WWD domain. Each residue of the *H. pylori* CcsBA WWD domain was individually mutated to cysteine. The single amino acid variants were affinity purified in groups, each with a WT control. Groups were analyzed by a) Coomassie total staining and b) α-GST immunoblotting. CcsBA purifies as two major polypeptides: GST:CcsB' and 'CcsA . ' – indicates natural proteolysis. *CcsA – proteolyzed polypeptide containing CcsA WWD domain.

GST* - proteolyzed GST. c) Heme stains were quantified by determining the ratio of CcsA bound heme to *b*-type (free) heme from membrane 1 and membrane 2. Variants were compared to a WT that was induced and purified at the same time. Variants with a ratio of >2 for CcsA bound to *b*-type heme were further assessed. d-h) To demonstrate the *CcsA heme-stained polypeptide is distinct from GST* the following were aligned by the MW markers. d) Coomassie to show total protein. Note, this Coomassie is also presented in Fig 4a. e) α-GST immunoblot. f, g) Heme stain. Note *b*-type (free) heme can transfer through a 0.2 μM nitrocellulose membrane, therefore 2 membranes are layered for all heme stains and heme from both membranes is quantified to determine the final ratio. Note, Membrane 1 alone is presented in Fig 4b for simplicity and Membranes 1 (f) and 2 (g) are presented here to demonstrate free heme transfer. h) Inverted image of the heme stain of Membrane 1 to demonstrate low-levels of heme stained bands in W827C and W836C.

Supplementary Figure 8

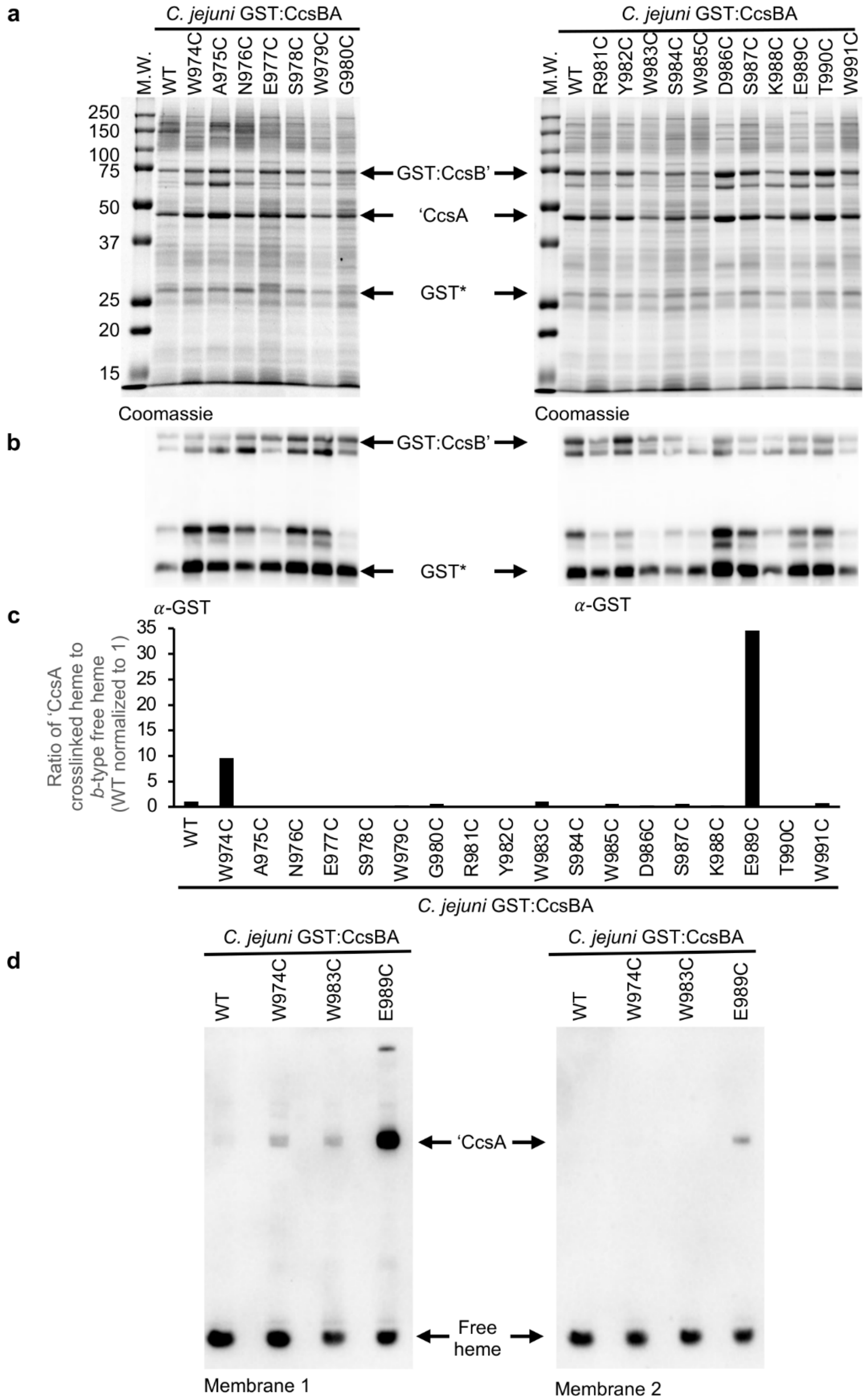

Supplementary Figure 8. Cysteine/heme crosslinking in the *C. jejuni* CcsBA WWD domain. Each residue of the *C. jejuni* CcsBA WWD domain was individually mutated to cysteine. The single amino acid variants were affinity purified in groups, each with a WT control. Groups were analyzed by a) Coomassie total staining and b) α-GST immunoblotting. CcsBA purifies as two major polypeptides: GST:CcsB' and 'CcsA . ' – indicates natural proteolysis. GST* - proteolyzed GST. c) Heme stains were quantified by determining the ratio of CcsA bound heme to *b*-type (free) heme from membrane 1 and membrane 2. Variants with a ratio of >2.0 for CcsA bound to *b*-type heme were further assessed. d) As described in

Supplementary Figure 7, heme from both membranes is quantified to determine the final ratio in these experiments. Note, Membrane 1 alone is presented in Fig 5b for simplicity and Membranes 1 and 2 are presented here to demonstrate free heme transfer.

Supplementary Figure 9

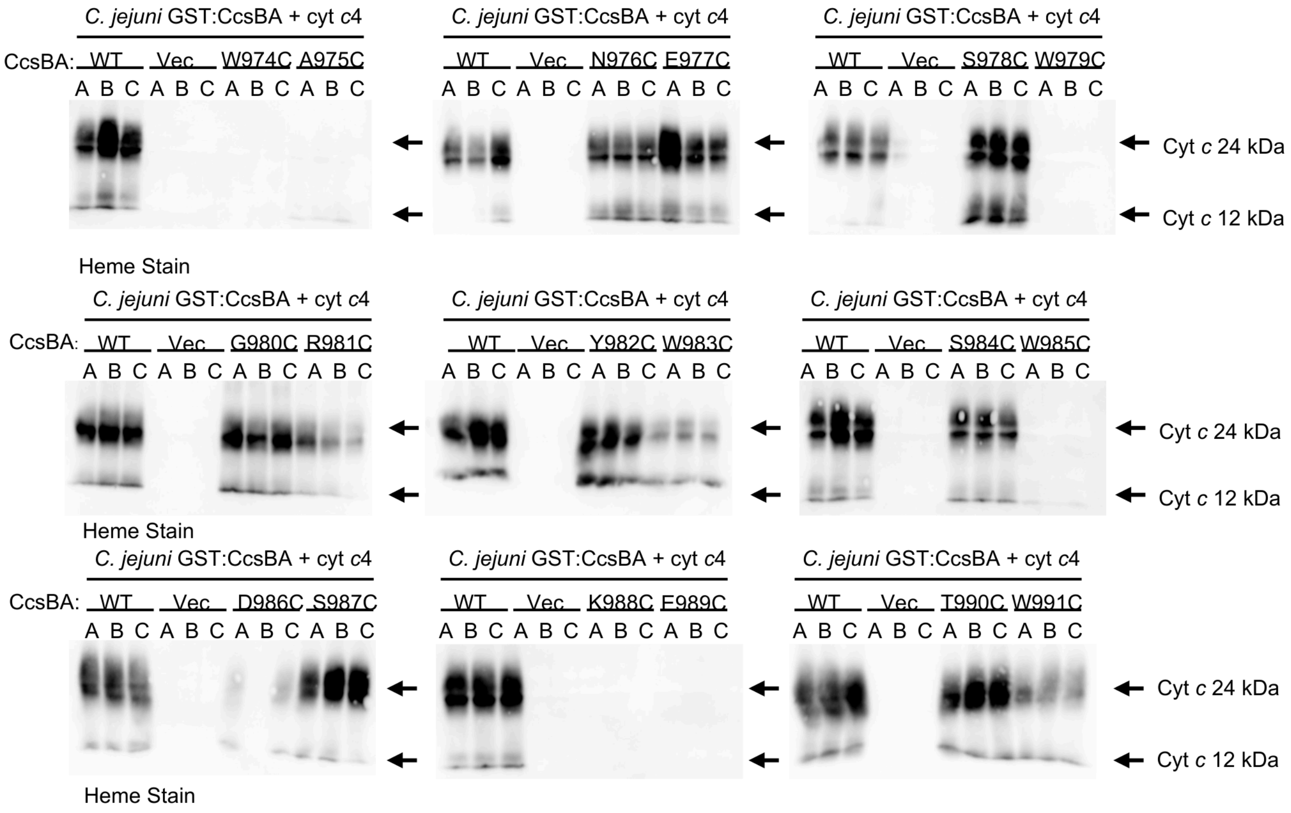

Supplementary Figure 9. Synthase function of *C. jejuni* CcsBA WWD cysteine variants. As described in Figure 5, the WWD variants were co-expressed with cytochrome c_4 in *E. coli Δccm* to determine synthase function. Representative triplicate experiment is shown. Each membrane/heme stain contains WT and vector controls for normalization during quantification. Heme stains were quantified to determine synthase function with WT normalized to 100%. Quantification is displayed in Figure 5h. Single samples of WT, vector and each cysteine variant were selected to generate Figure 5g.

Supplementary Figure 10

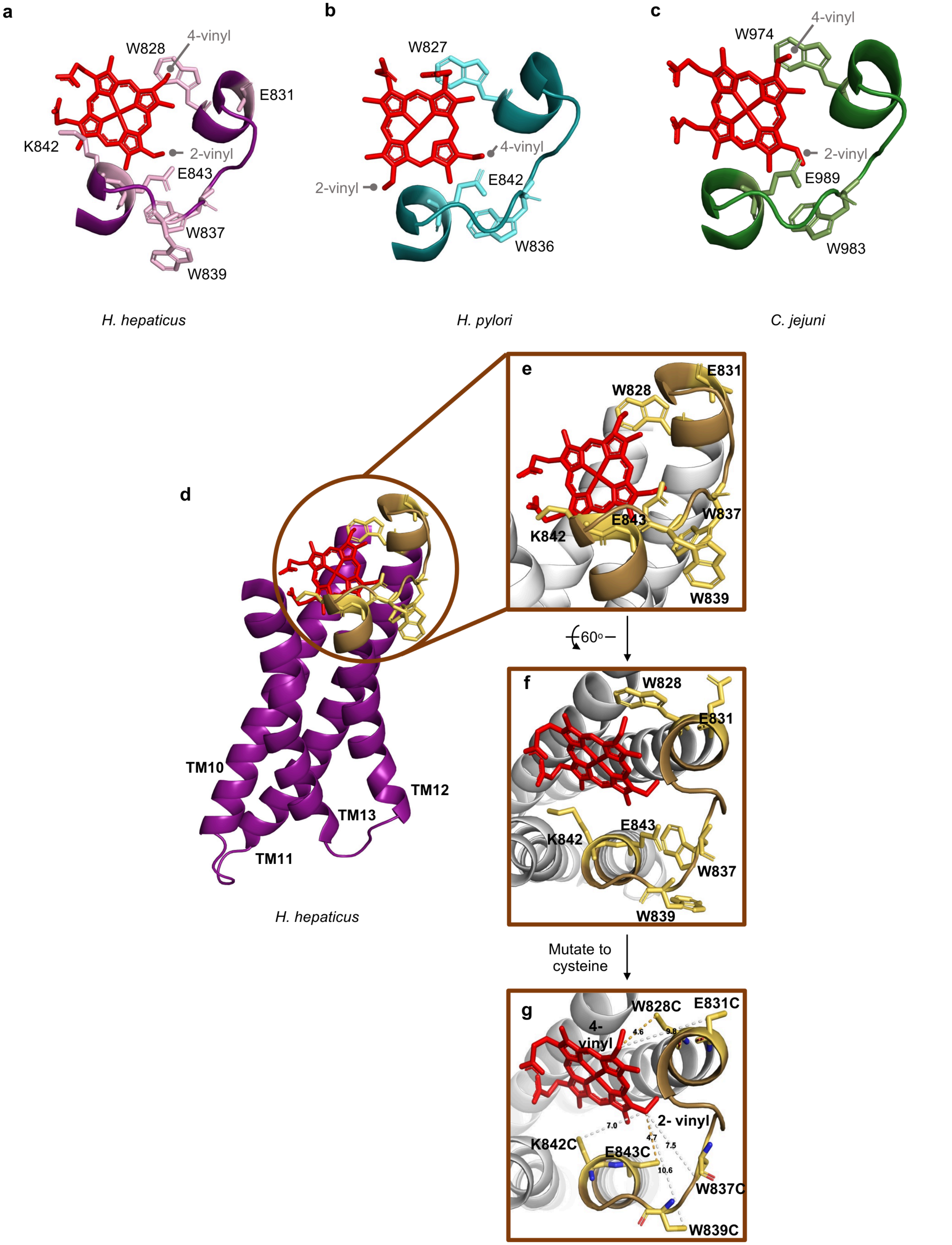

Supplementary Figure 10. **WWD domain-heme interaction is mediated by conserved residues**. a) The cryo-EM structure of *H. hepaticus* CcsBA WWD domain with heme (CcsBA deep purple, open, PDB

79SY[5]; heme red) demonstrates the vinyl groups (labeled in gray) in close proximity to cysteine/heme crosslinking residues (indicated by amino acid structure and labeled in black) b-c) and the AlphaFold 3[4] was utilized to predict the structures of b) *H. pylori* (deep teal) with heme (red). The predicted heme 4-vinyl is positioned near a cysteine/heme crosslinking residue, but the 2-vinyl is not. c) *C. jejuni* (forest green) with heme (red) prediction indicates the heme vinyl groups are positioned similarly to *H. hepaticus*. d) The WWD 'core region' of *H. hepaticus* CcsBA is shown with WWD domain colored in sand (open, PDB 79SY[5]). e) Zoomed in WWD domain with the six residues identified to form a cysteine/heme crosslink in *H. hepaticus* CcsBA[7] are displayed as sticks, heme is red. f) The WWD domain, rotated 60º around the X axis, provides better visualization of W828 and E843. g) The residues that form the cysteine/heme crosslink are shown with the predicted cysteine mutation. The relative bond length between the cysteine and the indicated heme vinyl group is shown with a dotted line. Bond lengths <5 Å are shown as a sand colored dotted line.

Supplementary Figure 11

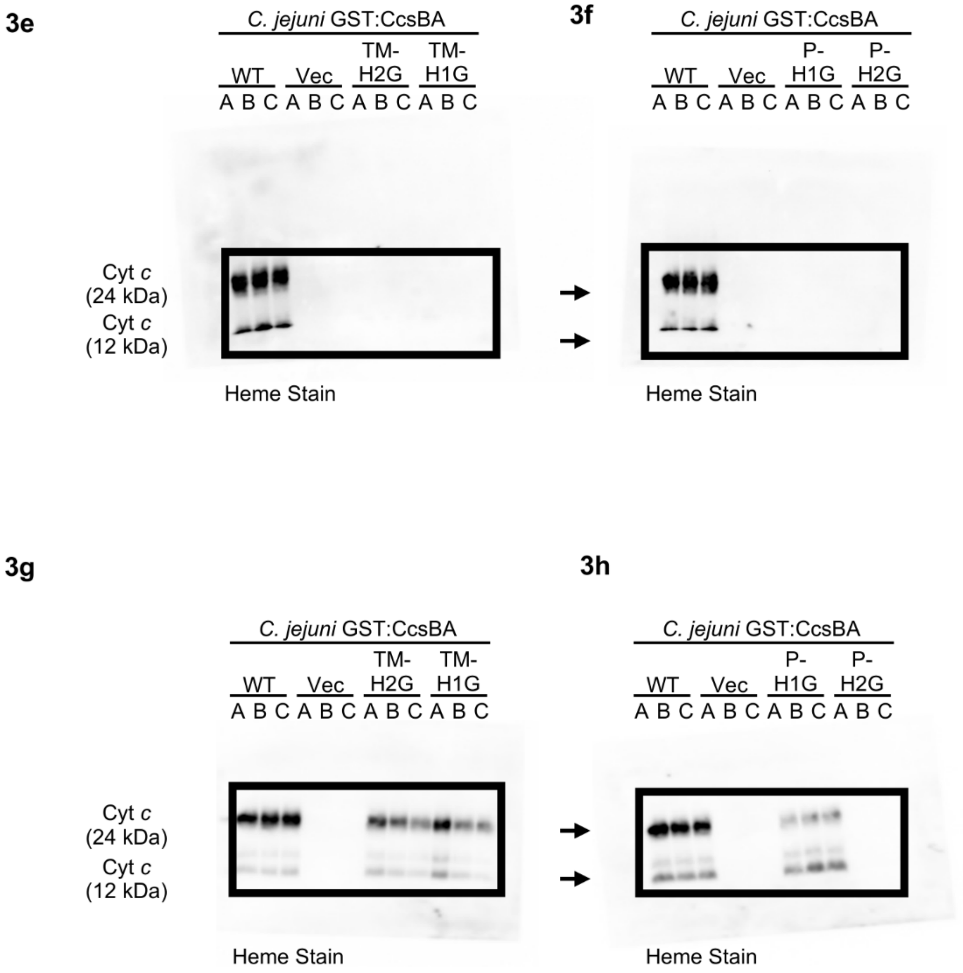

Supplementary Figure 11. Uncropped gel images for Figure 3. Black box indicates portion of gel displayed in figure. Relevant lanes are labeled.

Supplementary Figure 12

5g

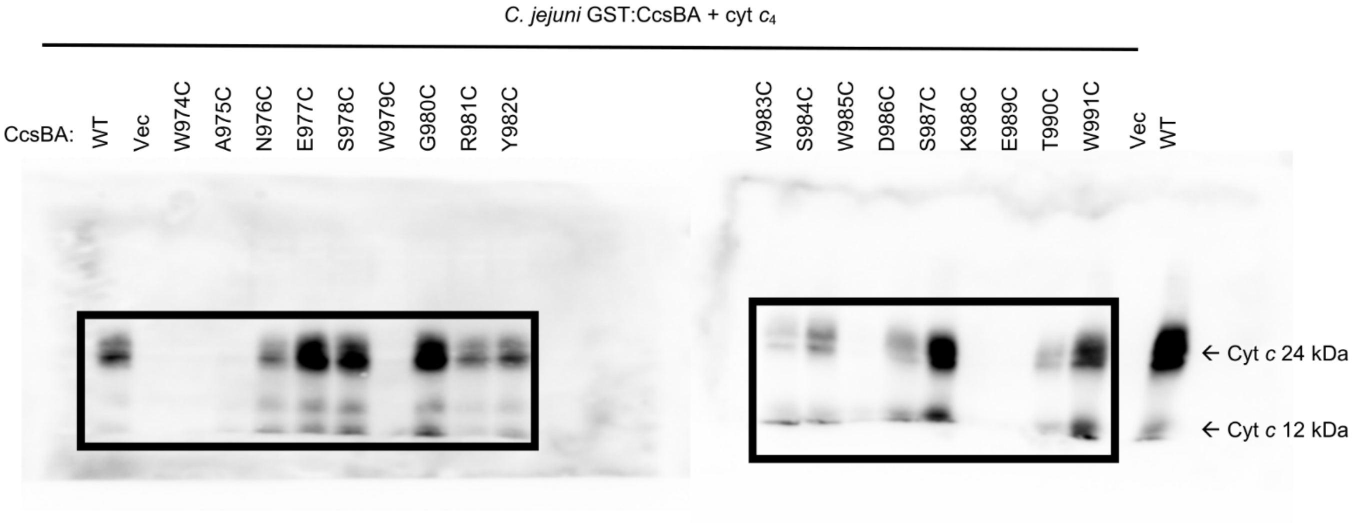

Supplementary Figure 12. Uncropped gel images for Figure 5. Black box indicates portion of gel displayed in figure. Relevant lanes are labeled.

Supplementary Methods

Construction of H. pylori and C. jejuni GST:CcsBA
pRGK334[10], *H. pylori* GST:CcsBA was determined to have an extension of genomic DNA after the CcsBA stop codon (AGGATAACCATGTTCCAACCCCTATTAGACGCG). This extension was removed via QuikChange II site-directed mutagenesis protocol utilizing primers MSP638/MSP639, resulting in pMCS1075. pMCS1075 was used a template for all His→Gly and WWD cysteine variants in this manuscript.

C. jejuni CcsBA was amplified from genomic DNA using the Cj forward and Cj reverse primers (see Supplementary Table 1). PCR product was digested with BamH1 and XhoI and ligated into a similarly digested pGEX4T-1 vector. Clone was confirmed by sequencing.

Construction of single amino acid substitutions
Single amino acid substitutions were constructed via QuikChange II site directed mutagenesis (Agilent technologies) according to the manufacturer's instructions. A complete list of strains, plasmids and primers for all cloning is provided in Supplementary Table 1.

Protein purifications
Affinity purifications of GST:CcsBA fusions were performed as previously described[7]. Briefly, *E. coli* strain C43 was used for recombinant protein expression. Overnight (saturated) starter cultures were diluted 1:200 in 1L LB with selective antibiotics, grown to saturation (~16-18 hours) at 24°C and 240 rpm, induced with 1 mM Isopropyl-beta-D-thiogalactoside (IPTG, GoldBio) for 6 hours, harvested by centrifugation and cell pellets were stored at -80ºC. Cell pellets were resuspended in Resin buffer (20 mM Tris pH8, 100 mM NaCl), supplemented with 1 mM phenylmethansulfonyl fluoride (PMSF, Sigma-Aldrich) for protease inhibition and 1 mg/mL egg white lysozyme (Goldbio). Cells were lysed via sonication (Branson250 sonicator), cell debris was cleared by centrifugation at 24000*g* for 1 hour, 4ºC. Soluble and membrane fractions were separated by high-speed ultracentrifugation for 45 minutes at 100000*g*, 4ºC. Membrane pellets were solubilized in Resin buffer supplemented with 1% n-dodecyl-β-d-maltopyranoside (DDM; Goldbio). Affinity purification by batch method with glutathione agarose (Pierce) was performed, resin was washed by gravity flow, eluted in 6 mL Resin buffer with 0.02% DDM and 20 mM L-glutathione (Sigma-Aldrich) and concentrated in a VivaspinTurbo4 100 kDa filter (Sartorius). Protein concentration was determined by Bradford Reagent (Sigma). Proteins were separated by SDS-PAGE and visualized by total protein stain with Coomassie Blue.

Functional (heme attachment) assays
The indicated CcsBA variants were co-expressed with cytochrome c_4:His (pRGK332) in strain C43 *Δccm*::Kan$^{R.}$ For *C. jejuni* CcsBA variants, saturated starter cultures were backdiluted 1:5 into 5 ml of LB with appropriate antibiotics and grown for 3 hours at 37°C and 200 rpm. Cells were induced with 0.1 mM IPTG and 0.2% arabinose and grown for additional 3 hours at 37°C and 200 rpm prior. Cells were collected by centrifugation at 3700 rpm for 10 minutes and cell pellets were stored at -80°C. Cell pellets were lysed with 200 µl Bacterial Protein Extraction Reagent (B-PER™, Thermo Scientific) per the manufacturer's instructions, including the addition of 2 µl 1M $MgCl_2$ and 0.4 DNase per tube with an hour shaking on ice at 100 rpm prior to centrifugation steps. 150 µg of B-PER cell lysates were separated by 12.5% SDS-PAGE and analyzed for synthase activity (i.e. formation of holocytochrome *c*) by heme staining.

Supplementary Table 1.

Supplementary Table 1. Strains, plasmids and primers employed in this study

Strain/ Plasmid	Description	Reference
E. coli		
NEB 5-α	fhuA2 Δ(argF-lacZ)U169 phoA glnV44 Φ80 Δ(lacZ)M15 gyrA96 recA1 relA1 endA1 thi-1 hsdR17	
MS36	C43 *Δccm::kanR*, deleted for all *ccm* genes	[7]
Plasmid		
pRGK332	pBAD cytochrome c4:His (*Bordetella pertussis*)	[10]
pRGK334	pGEX *H. pylori* GST:CcsBA	[10]
pMCS1075	pGEX *H. pylori* GST:CcsBA	This study
pMCS1059	pGEX *H. pylori* GST:CcsBA (W827C)	This study
pMCS1060	pGEX *H. pylori* GST:CcsBA (A828C)	This study
pMCS959	pGEX *H. pylori* GST:CcsBA (N829C)	This study
pMCS1062	pGEX *H. pylori* GST:CcsBA (E830C)	This study
pMCS957	pGEX *H. pylori* GST:CcsBA (S831C)	This study
pMCS990	pGEX *H. pylori* GST:CcsBA (W832C)	This study
pMCS991	pGEX *H. pylori* GST:CcsBA (G833C)	This study
pMCS958	pGEX *H. pylori* GST:CcsBA (R834C)	This study
pMCS960	pGEX *H. pylori* GST:CcsBA (Y835C)	This study
pMCS1064	pGEX *H. pylori* GST:CcsBA (W836C)	This study
pMCS961	pGEX *H. pylori* GST:CcsBA (G837C)	This study
pMCS1066	pGEX *H. pylori* GST:CcsBA (W838C)	This study
pMCS962	pGEX *H. pylori* GST:CcsBA (D839C)	This study
pMCS963	pGEX *H. pylori* GST:CcsBA (P840C)	This study
pMCS1067	pGEX *H. pylori* GST:CcsBA (K841C)	This study
pMCS1069	pGEX *H. pylori* GST:CcsBA (E842C)	This study
pMCS964	pGEX *H. pylori* GST:CcsBA (T843C)	This study
pMCS965	pGEX *H. pylori* GST:CcsBA (W844C)	This study
pMCS966	pGEX *H. pylori* GST:CcsBA (H86G)	This study
pMCS967	pGEX *H. pylori* GST:CcsBA (H857G)	This study
pMCS988	pGEX *H. pylori* GST:CcsBA (H760G)	This study
pMCS989	pGEX *H. pylori* GST:CcsBA (H896G)	This study
pMCS784	pGEX *C. jejuni* GST:CcsBA	This study
pMCS780	pGEX *C. jejuni* GST:CcsBA (W974C)	This study
pMCS879	pGEX *C. jejuni* GST:CcsBA (A975C)	This study
pMCS880	pGEX *C. jejuni* GST:CcsBA (N976C)	This study
pMCS782	pGEX *C. jejuni* GST:CcsBA (E977C)	This study
pMCS881	pGEX *C. jejuni* GST:CcsBA (S978C)	This study
pMCS882	pGEX *C. jejuni* GST:CcsBA (W979C)	This study
pMCS883	pGEX *C. jejuni* GST:CcsBA (G980C)	This study
pMCS884	pGEX *C. jejuni* GST:CcsBA (R981C)	This study
pMCS885	pGEX *C. jejuni* GST:CcsBA (Y982C)	This study
pMCS797	pGEX *C. jejuni* GST:CcsBA (W983C)	This study
pMCS886	pGEX *C. jejuni* GST:CcsBA (S984C)	This study
pMCS788	pGEX *C. jejuni* GST:CcsBA (W985C)	This study
pMCS887	pGEX *C. jejuni* GST:CcsBA (D986C)	This study

pMCS888	pGEX *C. jejuni* GST:CcsBA (S987C)	This study	
pMCS789	pGEX *C. jejuni* GST:CcsBA (K988C)	This study	
pMCS790	pGEX *C. jejuni* GST:CcsBA (E989C)	This study	
pMCS889	pGEX *C. jejuni* GST:CcsBA (T990C)	This study	
pMCS890	pGEX *C. jejuni* GST:CcsBA (W991C)	This study	
pMCS792	pGEX *C. jejuni* GST:CcsBA (H82G)	This study	
pMCS794	pGEX *C. jejuni* GST:CcsBA (H1004G)	This study	
pMCS795	pGEX *C. jejuni* GST:CcsBA (H1043G)	This study	
pMCS799	pGEX *C. jejuni* GST:CcsBA (H904G)	This study	

**Oligonucleotide**	**Sequence (5'-->3')**	**Purpose (Cloning for:)**	**Template**
Cj forward	gaggatccatgaaaaatataataaaaagcataggggat	pMCS784	**genomic**
Cj reverse	gtctcgagttaaagtttgcctacaaaatttcgcttcct	pMCS784	**genomic**
MSP0445	ggaaatttttaggtgcaatttgcgcaaatgaaagttgggggag	pMCS780	pMCS784
MSP0446	ctcccccaactttcatttgcgcaaattgcacctaaaaaatttcc	pMCS780	pMCS784
MSP0447	ggaaatttttaggtgcaatttgggcaaattgcagttgggggagatattgg	pMCS782	pMCS784
MSP0448	ccaatatctcccccaactgcaatttgcccaaattgcacctaaaaaatttcc	pMCS782	pMCS784
MSP0449	gggcaaatgaaagttgggggagatattgcagttgggattctaagg	pMCS797	pMCS784
MSP0450	ccttagaatcccaactgcaatatctcccccaactttcatttgccc	pMCS797	pMCS784
MSP0451	ggggagatattggagttgcgattctaaggaaacttgg	pMCS788	pMCS784
MSP0452	ccaagtttccttagaatcgcaactccaatatctcccc	pMCS788	pMCS784
MSP0453	ggagatattggagttgggattcttgcgaaacttgggctttagttagcattttgg	pMCS789	pMCS784
MSP0454	ccaaaatgctaactaaagcccaagtttcgcaagaatcccaactccaatatctcc	pMCS789	pMCS784
MSP0455	ggagatattggagttgggattctaagtgcacttgggctttagttagcattttgg	pMCS790	pMCS784
MSP0456	ccaaaatgctaactaaagcccaagtgcacttagaatcccaactccaatatctcc	pMCS790	pMCS784
MSP0457	gggcttaaaaaattacctttaatgatttttggtatttctttttatttattttagtggg	pMCS792	pMCS784
MSP0458	cccactaaaataaataaaaaagaaataccaaaaatcattaaaggtaattttttaagccc	pMCS792	pMCS784
MSP0459	gggctttagttagcattttggtttatgcagcaattttgggtcttagaatgattccaaaatattgtaatc	pMCS794	pMCS784
MSP0460	gattacaatattttggaatcattctaagacccaaaattgctgcataaaccaaaatgctaactaaagccc	pMCS794	pMCS784
MSP0461	cctgtgctcaattcttattggcttagtattggtgtatctgttattactgctagttatgg	pMCS799	pMCS784
MSP0462	ccataactagcagtaataacagatacaccaatactaagccaataagaattgagcacagg	pMCS799	pMCS784
MSP0463	ggagtaaattacttttaacaggacttggttcttacgctgcaggcg	pMCS795	pMCS784
MSP0464	cgcctgcagcgtaagaaccaagtcctgttaaaagtaatttactcc	pMCS795	pMCS784
MSP0507	ggaaatttttaggtgcaatttggtgcaatgaaagttggggg	pMCS879	pMCS784
MSP0508	cccccaactttcattgcaccaaattgcacctaaaaaatttcc	pMCS879	pMCS784
MSP0509	gaaatttttaggtgcaatttgggcatgtgaaagttgggggagatattg	pMCS880	pMCS784
MSP0510	caatatctcccccaactttcacatgcccaaattgcacctaaaaaatttc	pMCS880	pMCS784
MSP0511	ggtgcaatttgggcaaatgaatgttgggggagatattgg	pMCS881	pMCS784
MSP0512	ccaatatctcccccaacattcatttgcccaaattgcacc	pMCS881	pMCS784
MSP0513	gggcaaatgaaagttgcgggagatattggagttg	pMCS882	pMCS784
MSP0514	caactccaatatctcccgcaactttcatttgccc	pMCS882	pMCS784
MSP0515	gggcaaatgaaagttggtgcagatattggagttggg	pMCS883	pMCS784
MSP0516	cccaactccaatatctgcaccaactttcatttgccc	pMCS883	pMCS784
MSP0517	ggcaaatgaaagttgggggtgctattggagttgggattctaagg	pMCS884	pMCS784
MSP0518	ccttagaatcccaactccaatagcacccccaactttcatttgcc	pMCS884	pMCS784
MSP0519	ggcaaatgaaagttgggggagatgttggagttggg	pMCS885	pMCS784
MSP0520	cccaactccaacatctcccccaactttcatttgcc	pMCS885	pMCS784
MSP0521	ggagttgggggagatattggtgttgggattctaagg	pMCS886	pMCS784
MSP0522	ccttagaatcccaacaccaatatctcccccaactcc	pMCS886	pMCS784

MSP0523	ggggagatattggagttggtgttctaaggaaacttgggc	pMCS887	pMCS784
MSP0524	gcccaagtttccttagaacaccaactccaatatctcccc	pMCS887	pMCS784
MSP0525	gggggagatattggagttgggattgtaaggaaacttgggc	pMCS888	pMCS784
MSP0526	gcccaagtttccttacaatcccaactccaatatctccccc	pMCS888	pMCS784
MSP0527	ggagatattggagttgggattctaaggaatgttgggctttagttagc	pMCS889	pMCS784
MSP0528	gctaactaaagcccaacattccttagaatcccaactccaatatctcc	pMCS889	pMCS784
MSP0529	gggattctaaggaaacttgcgctttagttagcattttgg	pMCS890	pMCS784
MSP0530	ccaaaatgctaactaaagcgcaagtttccttagaatccc	pMCS890	pMCS784
MSP0531	cgccagcctttttttcggcagctccttgattttcatcattttagg	pMCS966	pMCS1075
MSP0532	cctaaaatgatgaaaatcaaggagctgccgaaaaaaaggctggcg	pMCS966	pMCS1075
MSP0533	gcgtctatgctttaatcttggggttgcgttttctaggctctcacaattgg	pMCS967	pMCS1075
MSP0534	caattgtgagagcctagaaaacgcaaccccaagattaaagcatagacgc	pMCS967	pMCS1075
MSP0535	ggtattaaaatcctattggctcaatatcggagtctctgtcattaccgc	pMCS988	pMCS1075
MSP0536	gcggtaatgacagagactccgatattgagccaataggattttaatacc	pMCS988	pMCS1075
MSP0537	ggcgtgaattactacctttctggcttggggagctatgccgcaggc	pMCS989	pMCS1075
MSP0538	gcctgcggcatagctccccaagccagaaaggtagtaattcacgcc	pMCS989	pMCS1075
MSP0555	gccgggaatttcttaggcggggtgtgcgcgaatgaatcttgggggcg	pMCS1059	pMCS1075
MSP0556	cgcccccaagattcattcgcgcacaccccgcctaagaaattcccggc	pMCS1059	pMCS1075
MSP0557	gccgggaatttcttaggcggggtgtggtgcaatgaatcttgggggcg	pMCS1060	pMCS1075
MSP0558	cgcccccaagattcattgcaccacaccccgcctaagaaattcccggc	pMCS1060	pMCS1075
MSP0559	ggcggggtgtgggcgtgtgaatcttgggggcg	pMCS959	pMCS1075
MSP0560	cgcccccaagattcacacgcccacaccccgcc	pMCS959	pMCS1075
MSP0561	ggcggggtgtgggcgaattgctcttgggggcgttattggggg	pMCS1062	pMCS1075
MSP0562	cccccaataacgcccccaagagcaattcgcccacaccccgcc	pMCS1062	pMCS1075
MSP0563	gggtgtgggcgaatgaatgttgggggcgttattggggg	pMCS957	pMCS1075
MSP0564	cccccaataacgcccccaacattcattcgcccacaccc	pMCS957	pMCS1075
MSP0565	gggcgaatgaatcttgcgggcgttattggggg	pMCS990	pMCS1075
MSP0566	cccccaataacgcccgcaagattcattcgccc	pMCS990	pMCS1075
MSP0567	ggcgaatgaatcttggtgccgttattgggggtggg	pMCS991	pMCS1075
MSP0568	cccacccccaataacggcaccaagattcattcgcc	pMCS991	pMCS1075
MSP0569	gggcgaatgaatcttgggggtgttattgggggtggg	pMCS958	pMCS1075
MSP0570	cccacccccaataacacccccaagattcattcgccc	pMCS958	pMCS1075
MSP0571	gggcgaatgaatcttgggggcgttgttgggggtggg	pMCS960	pMCS1075
MSP0572	cccacccccaacaacgcccccaagattcattcgccc	pMCS960	pMCS1075
MSP0573	gggggcgttattgcgggtgggaccc	pMCS1064	pMCS1075
MSP0574	gggtcccacccgcaataacgccccc	pMCS1064	pMCS1075
MSP0575	gggggcgttattggtgctgggaccctaaagaaacttgggc	pMCS961	pMCS1075
MSP0576	gcccaagtttctttagggtcccagcaccaataacgccccc	pMCS961	pMCS1075
MSP0577	ggcgttattgggggtgcgaccctaaagaaacttgggc	pMCS1066	pMCS1075
MSP0578	gcccaagtttctttagggtcgcacccccaataacgcc	pMCS1066	pMCS1075
MSP0579	ggcgttattgggggtggtgccctaaagaaacttgggc	pMCS962	pMCS1075
MSP0580	gcccaagtttctttagggcaccacccccaataacgcc	pMCS962	pMCS1075
MSP0581	ggcgttattgggggtgggactgtaaagaaacttgggc	pMCS963	pMCS1075
MSP0582	gcccaagtttctttacagtcccacccccaataacgcc	pMCS963	pMCS1075
MSP0583	gggggtgggacccttgcgaaacttgggcattgatttctatttgcg	pMCS1067	pMCS1075
MSP0584	cgcaaatagaaatcaatgcccaagtttcgcaagggtcccaccccc	pMCS1067	pMCS1075
MSP0585	gggaccctaaatgcacttgggcattgatttctatttgcg	pMCS1069	pMCS1075
MSP0586	cgcaaatagaaatcaatgcccaagtgcatttagggtccc	pMCS1069	pMCS1075
MSP0587	gggaccctaaagaatgttgggcattgatttctatttgcg	pMCS964	pMCS1075
MSP0588	cgcaaatagaaatcaatgcccaacattctttagggtccc	pMCS964	pMCS1075

MSP0589	gggaccctaaagaaacttgcgcattgatttctatttgcg	pMCS965	pMCS1075
MSP0590	cgcaaatagaaatcaatgcgcaagtttctttagggtccc	pMCS965	pMCS1075
MSP0638	cgccatttgagtttgcctaaattagcttaagaattcatcgtgactgactgacg	pMCS1075	pRGK334
MSP0639	cgtcagtcagtcacgatgaattcttaagctaatttaggcaaactcaaatggcg	pMCS1075	pRGK334

Supplementary References

1. Di Tommaso, P. *et al.* T-Coffee: a web server for the multiple sequence alignment of protein and RNA sequences using structural information and homology extension. *Nucleic Acids Research* **39**, W13–W17 (2011).
2. Notredame, C., Higgins, D. G. & Heringa, J. T-coffee: a novel method for fast and accurate multiple sequence alignment 1 1Edited by J. Thornton. *Journal of Molecular Biology* **302**, 205–217 (2000).
3. Robert, X. & Gouet, P. Deciphering key features in protein structures with the new ENDscript server. *Nucleic Acids Research* **42**, W320–W324 (2014).
4. Abramson, J. *et al.* Accurate structure prediction of biomolecular interactions with AlphaFold 3. *Nature* **630**, 493–500 (2024).
5. Mendez, D. L. *et al.* Cryo-EM of CcsBA reveals the basis for cytochrome c biogenesis and heme transport. *Nat Chem Biol* **18**, 101–108 (2022).
6. Krogh, A., Larsson, B., von Heijne, G. & Sonnhammer, E. L. L. Predicting transmembrane protein topology with a hidden markov model: application to complete genomes11Edited by F. Cohen. *Journal of Molecular Biology* **305**, 567–580 (2001).
7. Sutherland, M. C. *et al.* Structure-Function Analysis of the Bifunctional CcsBA Heme Exporter and Cytochrome *c* Synthetase. *mBio* **9**, e02134-18 (2018).
8. Grunow, A. L., Carroll, S. C., Kreiman, A. N. & Sutherland, M. C. Structure-function analysis of the heme-binding WWD domain in the bacterial holocytochrome *c* synthase, CcmFH. *mBio* e01509-23 (2023) doi:10.1128/mbio.01509-23.
9. Sutherland, M. C., Jarodsky, J. M., Ovchinnikov, S., Baker, D. & Kranz, R. G. Structurally Mapping Endogenous Heme in the CcmCDE Membrane Complex for Cytochrome c Biogenesis. *J. Mol. Biol.* **430**, 1065–1080 (2018).
10. Feissner, R. E. *et al.* Recombinant cytochromes c biogenesis systems I and II and analysis of haem delivery pathways in Escherichia coli. *Mol. Microbiol.* **60**, 563–577 (2006).
