# Supplementary material for: Helicobacter pylori and Campylobacter jejuni bacterial holocytochrome c synthase structure-function analysis reveals conservation of heme binding
Source: Commun Biol. 2024 Aug 13;7:984. doi: 10.1038/s42003-024-06688-3 (PMC11322641; doi:10.1038/s42003-024-06688-3)
Supplement: Supplementary file 3 — Description of additional supplementary files [file 42003_2024_6688_MOESM3_ESM.pdf]

Description of Additional Supplementary Files

File name: Supplementary data 1

Description: Source data for graphs presented in Fig. 3i, j, k, l

File name: Supplementary data 2

Description: Source data for graph presented in Fig. 5h
