# Supplementary material for: Helicobacter pylori and Campylobacter jejuni bacterial holocytochrome c synthase structure-function analysis reveals conservation of heme binding
Source: Commun Biol. 2024 Aug 13;7:984. doi: 10.1038/s42003-024-06688-3 (PMC11322641; doi:10.1038/s42003-024-06688-3)
Supplement: Supplementary file 6 — Reporting Summary [file 42003_2024_6688_MOESM6_ESM.pdf]

nature portfolio

Corresponding author(s): Molly C. Sutherland, corresponding author for "Helicobacter pylori and Campylobacter jejuni bacterial holocytochrome c synthase structure-function analysis reveals conservation of heme binding"

Last updated by author(s): July 22, 2024

Reporting Summary

Nature Portfolio wishes to improve the reproducibility of the work that we publish. This form provides structure for consistency and transparency in reporting. For further information on Nature Portfolio policies, see our Editorial Policies and the Editorial Policy Checklist.

Statistics

For all statistical analyses, confirm that the following items are present in the figure legend, table legend, main text, or Methods section.

n/a	Confirmed	
☒	☐	The exact sample size (*n*) for each experimental group/condition, given as a discrete number and unit of measurement
☐	☒	A statement on whether measurements were taken from distinct samples or whether the same sample was measured repeatedly
☒	☐	The statistical test(s) used AND whether they are one- or two-sided<br>*Only common tests should be described solely by name; describe more complex techniques in the Methods section.*
☒	☐	A description of all covariates tested
☒	☐	A description of any assumptions or corrections, such as tests of normality and adjustment for multiple comparisons
☐	☒	A full description of the statistical parameters including central tendency (e.g. means) or other basic estimates (e.g. regression coefficient) AND variation (e.g. standard deviation) or associated estimates of uncertainty (e.g. confidence intervals)
☒	☐	For null hypothesis testing, the test statistic (e.g. F, t, r) with confidence intervals, effect sizes, degrees of freedom and P value noted<br>*Give P values as exact values whenever suitable.*
☒	☐	For Bayesian analysis, information on the choice of priors and Markov chain Monte Carlo settings
☒	☐	For hierarchical and complex designs, identification of the appropriate level for tests and full reporting of outcomes
☒	☐	Estimates of effect sizes (e.g. Cohen's d, Pearson's r), indicating how they were calculated

Our web collection on statistics for biologists contains articles on many of the points above.

Software and code

Policy information about availability of computer code

Data collection	Western blot and heme stain imaging Azure Biosystems Sapphire Biomolecular Imager; UV-vis spectroscopy: Shimadzu UV-1900i
Data analysis	AlphaFold protein modeling: AlphaFold 3 launched on 2024-05; Western blot and heme stain quantification: Azure Biosystems Azure Spot v.2.2.167; UV-vis spectroscopy - Shimadzu LabSolutions UV

For manuscripts utilizing custom algorithms or software that are central to the research but not yet described in published literature, software must be made available to editors and reviewers. We strongly encourage code deposition in a community repository (e.g. GitHub). See the Nature Portfolio guidelines for submitting code & software for further information.

Data

Policy information about availability of data

All manuscripts must include a data availability statement. This statement should provide the following information, where applicable:

- Accession codes, unique identifiers, or web links for publicly available datasets
- A description of any restrictions on data availability
- For clinical datasets or third party data, please ensure that the statement adheres to our policy

Uncropped gel images are provided in Supplementary Fig. 11 and 12.
Source Data for Fig. 3 and 5 are provided in Supplementary Data 1 and 2

Research involving human participants, their data, or biological material

Policy information about studies with human participants or human data. See also policy information about sex, gender (identity/presentation), and sexual orientation and race, ethnicity and racism.

Reporting on sex and gender	n/a
Reporting on race, ethnicity, or other socially relevant groupings	n/a
Population characteristics	n/a
Recruitment	n/a
Ethics oversight	n/a

Note that full information on the approval of the study protocol must also be provided in the manuscript.

Field-specific reporting

Please select the one below that is the best fit for your research. If you are not sure, read the appropriate sections before making your selection.

☒ Life sciences ☐ Behavioural & social sciences ☐ Ecological, evolutionary & environmental sciences

For a reference copy of the document with all sections, see nature.com/documents/nr-reporting-summary-flat.pdf

Life sciences study design

All studies must disclose on these points even when the disclosure is negative.

Sample size	Not relevant to this study - independent, biological replicates were performed.
Data exclusions	No data exclusions
Replication	Information on replicates is included in the figure legends. Generally, all data based on protein purifications (Coomassie, heme stain, western, UV-vis spectra) are representative of at least 3, independent purifications. Cytochrome c biogenesis functional assays were done with three, independent biological replicates that each contain 3 technical replicates. A representative biological replicate is included in the manuscript.
Randomization	Not relevant to this study.
Blinding	Not relevant to this study.

Reporting for specific materials, systems and methods

We require information from authors about some types of materials, experimental systems and methods used in many studies. Here, indicate whether each material, system or method listed is relevant to your study. If you are not sure if a list item applies to your research, read the appropriate section before selecting a response.

Materials & experimental systems

n/a	Involved in the study	
☐	☒	Antibodies
☒	☐	Eukaryotic cell lines
☒	☐	Palaeontology and archaeology
☒	☐	Animals and other organisms
☒	☐	Clinical data
☒	☐	Dual use research of concern
☒	☐	Plants

Methods

n/a	Involved in the study	
☒	☐	ChIP-seq
☒	☐	Flow cytometry
☒	☐	MRI-based neuroimaging

Antibodies

Antibodies used	α-GST - Invitrogen, PA1-982A
Validation	α-GST Invitrogen, PA1-982A - Polyclonal Rabbit/IgG. Detects glutathione-S-transferase (GST). Manufacturer applications - western blot and Immunuprecipitation

Plants

Seed stocks	n/a
Novel plant genotypes	n/a
Authentication	n/a
